# Comparative effectiveness of multiple androgen receptor signaling inhibitor medicines with androgen deprivation therapy for metastatic hormone-sensitive prostate cancer: a study in the real world

**DOI:** 10.3389/fonc.2024.1324181

**Published:** 2024-04-18

**Authors:** Yutong Lu, Jingqi Jiang, Gaoyang Yang, Hui Ding, Qihui Zheng, Luhua Ji, Yuhan Wang, Zhilong Dong, Zhenxing Zhai, Junqiang Tian, Yunxing Zhang, Juan Wang, Li Yang, Zhiping Wang

**Affiliations:** Department of Urology, Gansu Provincial Key Laboratory of Urological Disease Research, The Second Hospital of Lanzhou University, Lanzhou, Gansu, China

**Keywords:** metastatic hormone-sensitive prostate cancer, androgen deprivation therapy (ADT), prostate-specific antigen (PSA), androgen receptor signaling inhibitors medicines, overall survival (OS), disease progression

## Abstract

**Background:**

The current treatment strategy for metastatic Hormone-Sensitive Prostate Cancer (mHSPC) is the combination of Androgen Receptor Signaling Inhibitors (ARSIs) medicines with androgen deprivation therapy (ADT). However, there is a lack of real-world data comparing the efficacy of different ARSI pharmaceuticals. Therefore, the objective of this study was to compare the effectiveness and safety of bicalutamide, abiraterone, enzalutamide, and apalutamide in combination with ADT for patients with mHSPC.

**Methods:**

We retrospectively analyzed 82 patients diagnosed with mHSPC, including 18 patients treated with abiraterone acetate with prednisone, 21 patients with enzalutamide, 20 patients with apalutamide, and 23 patients with bicalutamide. We evaluated PSA progression-free survival (PSA-PFS), imaging progression-free survival (r PFS), castration resistance progression-free survival (CRPC-PFS), and overall survival (OS) using Kaplan-Meier survival analyses. Additionally, we explored relevant factors affecting prognosis through univariate and multivariate Cox risk-proportionality models. PSA response rates at 3, 6, and 12 months, nadir PSA levels (nPSA), and time to nadir (TTN) in different medication subgroups after treatment were documented, and we used one-way ANOVA to determine the effect of these measures on patient prognosis.

**Results:**

In comparison with bicalutamide, both enzalutamide and apalutamide have shown significant advantages in delaying disease progression among mHSPC patients. Specifically, enzalutamide has been found to significantly prolong PSA-PFS (HR 2.244; 95% CI 1.366-3.685, p=0.001), rPFS (HR 2.539; 95% CI 1.181-5.461; p= 0.007), CRPC-PFS (HR 2.131; 95% CI 1.295-3.506; p= 0.003), and OS (HR 2.06; 95% CI 1.183-3.585; P=0.005). Similarly, apalutamide has significantly extended PSA-PFS (HR 5.071; 95% CI 1.711-15.032; P= 0.003) and CRPC-PFS (HR 6.724; 95% CI 1.976-22.878; P=0.002) among patients. On the other hand, the use of abiraterone in combination with ADT did not demonstrate a significant advantage in delaying diseases progression when compared with the other three agents in mHSPC patients. There were no significant differences in overall adverse event rates among the four pharmaceuticals in terms of safety. Additionally, the observation of PSA kinetics revealed that enzalutamide, apalutamide, and abiraterone acetate had a significant advantage in achieving deep PSA response (PSA ≤ 0.2 ng/ml) compared with bicalutamide (p=0.007 at 12 months). Enzalutamide and apalutamide exhibited preeminence efficacy, with no substantial difference observed between the two medications.

**Conclusions:**

Abiraterone, enzalutamide, and apalutamide were found to significantly reduce and stabilize PSA levels in mHSPC patients more quickly and thoroughly than bicalutamide. Furthermore, enzalutamide and apalutamide were found to significantly prolong survival and delay disease progression in mHSPC patients compared with bicalutamide. It should be noted that abiraterone did not demonstrate a significant advantage in delaying disease compared with enzalutamide and apalutamide. After conducting drug toxicity analyses, it was determined that there were no significant differences among the four drugs.

## Introduction

Metastatic Hormone-Sensitive Prostate Cancer (mHSPC) is a highly aggressive disease that can affect multiple organs and systems in the body, leading to a poor prognosis (1). The primary objective of treatment is to slow down the progression of the disease and extend the survival of patients. Currently, the standard treatment for mHSPC involves androgen deprivation therapy (ADT), which has significantly improved the prognosis for patients with low-risk mHSPC. However, in patients with high-risk mHSPC, ADT monotherapy is not effective in reducing the risk of progression to CRPC and may increase the likelihood of adverse events such as bone pain and pathological fractures (2). Therefore, the addition of new medications to ADT therapy has emerged as an important approach to delay disease progression and enhance patient survival (3).

The combination of ADT with ARSIs medications or docetaxel chemotherapy has demonstrated efficacy in prolonging the time to disease progression and enhancing overall survival (OS) in patients diagnosed with mHSPC (4–6). Furthermore, in the comparison of docetaxel and ARSI medications for the treatment of mHSPC in conjunction with androgen deprivation therapy (ADT), no statistically significant differences were observed in terms of OS and medication toxicity (7). Nevertheless, it is important to note that differences in treatment modalities and individual patient characteristics (including factors such as the presence of myelosuppression risk in patients, personal selection preferences, and toxicity profiles) for intravenous administration make the clinical choice of docetaxel somewhat limited (8). Two recent randomized controlled trials have shown that the use of triple therapy (docetaxel + ARSIs + ADT) significantly improves overall survival in patients with mHSPC when compared to docetaxel combined with ADT (9, 10). However, triple therapy is not currently used in clinical practice to treat the full spectrum of mHSPC patients due to insufficient evidence from clinical trials and controversy over therapeutic goals (11). Instead, contemporary mHSPC treatment strategies rely on doublet therapy using ARSIs or chemotherapeutic agents in combination with ADT treatment. Triple therapy is only employed to improve prognosis in patients with (high volume) mHSPC (12).

Considering the limitations of docetaxel, the adoption of dual therapy combining ARSIs medications and ADT undoubtedly represents a more favorable therapeutic strategy for managing mHSPC. However, it is worth noting that current clinical studies primarily focus on comparing the efficacy of docetaxel and ARSIs in cross-sectional studies. There remains a research gap concerning the relative efficacy of different ARSIs pharmaceuticals and safety of the four drugs in patients with mHSPC. Hence, through the collection of information and follow-up data from a cohort of mHSPC patients undergoing treatment with enzalutamide, apalutamide, abiraterone acetate, and bicalutamide in a real-world clinical setting, our study aims to comprehensively compare and analyze the treatment profiles of patients receiving these various medications. Specifically, the study will evaluate parameters including pre-treatment PSA response, long-term imaging changes, disease progression status, and drug-related adverse events, in order to assess and discern differences in efficacy and safety among the four drugs in mHSPC patients.

## Patients and methods

### Data collection

Eighty-two patients diagnosed with mHSPC at the Second Hospital of Lanzhou University between 2018 and 2023 were retrospectively included in this study. These included 18 patients treated with abiraterone acetate (1000 mg per day) combined with prednisone (10 mg per day), 21 patients treated with enzalutamide (160 mg per day), 20 patients treated with apalutamide (240 mg per day), and 23 patients treated with bicalutamide (50 mg per day). All patients were treated with ADT (including LHRH agonists or orchiectomy). Patients who received other treatment modalities (including docetaxel combination) during the treatment period were excluded.

PSA levels, PSA response rates at 3, 6, and 12 months after the first dose of the medicine, PSA decline to nadir (nPSA), and time to PSA nadir (TTN) were recorded. Data were also collected on adverse events, PSA progression-free survival (PSA-PFS), radiographic progression-free survival (rPFS), CRPC progression-free survival (CRPC-PFS), and overall survival (OS) of patients in the four different subgroups. Computed tomography scans were used to assess visceral and lymph node metastases, whole-body Bone Scan were used to assess bone metastases, and pathological findings from puncture biopsies and imaging data were used to confirm the patient’s diagnosis. Adverse events were classified according to the International Cancer Centre Common Terminology for Adverse Events version 5.10 (CTCAE).

This study was approved by the Ethics Committee of the Second Hospital of Lanzhou University (Project No.2022A-101). According to the Helsinki Declaration of Principles, given the retrospective study design, informed consent was not required for this study.

### Assessment and follow-up

The primary endpoint was PSA-PFS, with time to PSA progression defined as the time from first dose to PSA progression according to Prostate Cancer Clinical Trials Working Group (PCWG2) criteria (13). Secondary endpoints were OS, rPFS, CRPC-PFS, nPSA, TTN, rate of PSA90 at 3 months post-dose and PSA levels at 3, 6, and 12months post-dose. OS was defined as the time from the first dose to death from any cause, and rPFS was defined as the time from the first dose to the first radiographic new lesion or confirmatory bone scan if bone metastases were detected. CRPC-PFS was defined as the time from the first dose to radiographic disease progression, PSA progression according to PCWG2 criteria, or symptomatic skeletal event, whichever occurred first. TTN is the time taken for PSA to fall to its nadir after treatment and nPSA is defined as the PSA level at the nadir of PSA after treatment. PSA90 is defined as a 90% reduction in PSA relative to baseline at 3 months after treatment. We will report PSA levels at the important time points of 3, 6, and 12 months after medications treatment.

### Statistical analysis

Differences in PSA-PFS, rPFS, CRPC-PFS, and OS were evaluated using the Kaplan-Meier method and the logrank test, and comparisons of efficacy were made between groups. Hazard ratio (HR) calculations were performed using the COX risk model, and univariate analyses were followed by COX multivariate analyses to analyze relevant indicators affecting patient prognosis. In addition, we will analyze the effect of nPSA and TTN on the prognosis of patients in each group using the chi-squared test and COX risk model. IBM SPSS Statistics version 26 (IBM Corp.) was used for statistical analysis. The significance level was set at p<0.05.

### Sample size calculation

This study employs a survival analysis design to investigate the impact of four drugs on the overall survival (OS) prognosis of patients with metastatic prostate cancer, using Cox regression analysis. The main evaluation indexes for observation were different drug treatment groupings. The results of the preexperiment showed that, compared to the bicalutamide group, the average HR of the remaining three drug groups was 0.632 times. Using PASS 15 software, the required number of cases was calculated to be 99. Taking into account the loss of visits and refusal of visits in 10% of cases, the final number was calculated to be at least 110.

## Result

### The patient baseline data

Apart from age and visceral metastases, there were no significant differences in other baseline characteristics between the four groups, with the majority of patients having a PSA greater than 100 ng/ml at initial diagnosis and more than 75% of patients having a Gleason score of 8 or greater. The age of the patients clustered around 70 years, with patients in the bicalutamide group being older than the other three groups (**Table 1**, 71.3 vs. 68.28 vs. 67.33 vs. 70.16, P=0.019). According to the CHAARTED criteria (14), approximately 56% of patients had high-volume disease. In terms of visceral metastases, patients in the enzalutamide and apalutamide treated groups were significantly better off than those in the other two groups (p=0.013). In addition, according to the LATITUDE risk criteria (15), about 58%t of patients had high-risk diseases, which was essentially similar in the four groups. (PSA levels less than 0.06 ng/ml were recorded as 0.06 ng/ml and greater than 100 ng/ml as 100 ng/ml).

**Table 1:** Clinical baseline characteristics.

	ABI	ENZ	APA	Bica	t/Z/c2value	*P* value
	(n=18)	(n=21)	(n=20)	(n=23)		
**Age**	68.28 ± 10.07	67.33 ± 8.05	71.30 ± 9.72	70.16 ± 9.00	9.96	0.019
**BMI**	22.34 ± 2.50	24.75 ± 2.95	23.68 ± 2.56	23.4 ± 2.86	2.51	0.065
**Gleason**					5.961	0.114
≤7	2 (11.1)	3 (14.3)	4 (20.0)	9 (39.1)		
≥8	16 (88.9)	18 (85.7)	16 (80.0)	14 (60.9)		
**ECOG**					5.24	0.155
0~1	14 (77.8)	18 (85.7)	14 (70.0)	11 (47.8)		
2	4 (22.2)	3 (14.3)	6 (30.0)	12 (53.2)		
**Hypertensive**	6 (33.3)	5 (23.8)	8 (40.0)	8 (34.78)	1.281	0.734
**Diabetes**	2 (11.1)	2 (9.5)	3 (15.0)	7 (30.4)	4.258	0.235
**Electrocardiogram**	3 (16.7)	4 (19.0)	3 (15.0)	3 (13.0)	0.428	0.934
PSA at diagnosis*(ng/ml)	100.00 (57.33-100)	100.00 (29.50-100)	100.00 (66.70-100)	100 (67.6-100)	2.075	0.501
**Systemic metastases**						
Bone	17 (94.4)	17 (81.0)	18 (90.0)	23 (100)	6.658	0.114
Visceral	0 (0.0)	4 (19.0)	4 (20.0)	0 (0.0)	11.963	0.013
Lymphatic-node	12 (66.7)	15 (71.4)	16 (80.0)	16 (69.6)	0.959	0.811
**Tumor volume**					0.504	0.917
high	9 (50.0)	11 (52.4)	12 (60.0)	14 (60.90)		
low	9 (50.0)	10 (47.6)	8 (40.0)	9 (39.10)		
**Disease risk**					0.726	0.612
high	9 (50.0)	12 (57.1)	11 (55.0)	16 (69.6)		
low	9 (50.0)	9 (42.9)	9 (45.0)	7 (33.3)		
**Radical resection during treatment**	2 (11.1)	1 (4.8)	0 (0.0)	4 (17.4)	4.712	0.867
The history of treatment						
Docetaxel	2 (11.1)	1 (4.8)	3 (15.0)	2 (8.7)	1.286	0.732
Radiotherapy	1 (5.6)	1 (4.8)	2 (10.0)	2 (0)	2.329	0.507
Duration of treatment(month)	36.867 ± 1.095	38.452 ± 1.1396	40.647 ± 1.583	42.559 ± 3.043	1.569	0.412

ABI: abiraterone, ENZ: enzalutamide, APA: apalutamide, Bica: bicalutamide. * :In Gansu, China, the upper limit for PSA testing is 100ng/ml from 2018 to 2022 due to limitations in testing technology and level. Therefore, if a patient’s PSA is greater than 100ng/ml at the time of initial treatment, it will also be considered as 100ng/ml.

### Survival outcome

Patients were enrolled and followed from January 2018 to September 2023, a total of 42.68% (35/82) of patients had PSA progression, 28.04% (23/82) had imaging progression, 42.68% (35/82) had castration resistance and 41.46% (34/82) had died (**Table 2**). There were significant differences between the four groups in terms of PSA-PFS (66.7% vs 71.4% vs 80.0% vs 17.4%, p=0.001), rPFS (66.7% vs 81.0% vs 90.0% vs 52.2%, p=0.015), CRPC-PFS (50.0% vs 76.2% vs 85.0% vs 21.7%, p=0.039) and OS (44.4% vs 81% vs 85% vs 26.1%, p=0.039) (**Table 3** and **Table 6** appendix). Compared with bicalutamide, enzalutamide and apalutamide significantly prolonged PSA-PFS in patients (HR 2.244, 95% CI 1.366-3.685, p=0.001; HR 5.071, 95% CI 1.71115.032, p=0.003), while abiraterone had no significant advantage in prolonging patients’ PSA-PFS compared with the remaining three groups. There was a significant benefit of enzalutamide (HR 2.131; 95% CI 1.295-3.506; P=0.003) and apalutamide (HR 6.724; 95% CI 1.976-22.878; P=0.002) in prolonging CRPC-PFS in patients compared with bicalutamide, whereas there was no significant difference between abiraterone and bicalutamide.

**Table 2:** Survival outcome.

	ABI (n=18)		ENZ (n=21)		APA (n=20)		Bica (n=23)		*P* value
	events (n%)	media time (months)	events (n%)	media time (months)	events (n%)	media time (months)	events (n%)	media time (months)	
**PSA-PFS**		NR		NR		NR	12.0 (8.08-15.92)		**0.001**
yes	12 (66.67)		15 (71.40)		16 (80.00)		4 (17.40)		
no	6 (33.33)		6 (28.60)		4 (20.00)		19 (82.60)		
**rPFS**		NR		NR		NR		NR	**0.015**
yes	12 (66.67)		17 (81.00)		18 (90.00)		12 (52.20)		
no	6 (33.33)		4 (19.00)		2 (10.00)		11 (47.80)		
**OS**	33 (23.7-42.3)			NR		NR	27 (21.10-32.9)		**0.039**
yes	8 (44.40)		17 (81)		17 (85.00)		6 (26.10)		
no	10 (65.60)		4 (19.00)		3 (15.00)		17 (73.90)		
**CRPC-PFS**		24 (18.27-29.73)		NR		NR	18.00 (12.37-23,64)		**0.001**
yes	9 (50.00)		16 (76.20)		17 (85.00)		5 (21.70)		
no	9 (50.00)		5 (23.80)		3 (15.00)		18 (78.30)		

Bold values represent statistically significant p-values.

NR means survival time not reaching median survival time.

**Table 3:** COX analysis.

Multifactor analysis												
PSA-PFS				rPFS			CRPC-PFS			OS		
	*P* value	HR	95%CI	*P* value	HR	95%CI	*P* value	HR	95%CI	*P* value	HR	95%CI
Group **0.041**	**0.041**	1.107	0.712-1.724	**0.043**	0.469	0.218-1.009	0.063	1.115	0.685-1.814	**0.016**	0.541	0.329-0.89
PSA 0.791	0.791	0.998	0.983-1.013	**0.08**	1.031	0.996-1.067	0.18	1.012	0.994-1.03	0.123	1.014	0.996-1.031
Gleason 0.882	0.882	1.089	0.351-3.384	0.061	0.103	0.009-1.113	0.421	0.625	0.199-1.965	**0.007**	0.163	0.043-0.611
Disease risk 0.718	0.718	1.265	0.352-4.545	0.862	1.201	0.153-9.409	0.222	0.432	0.113-1.659	0.135	0.344	0.085-1.390
Tumor volume 0.831	0.831	0.878	0.266-2.901	0.586	1.631	0.281-9.467	0.143	2.499	0.734-8.509	0.583	1.43	0.398-5.139
Systemic metastases												
Lymphaticnode	0.72	0.839	0.321-2.192	0.212	2.33	0.617-8.799	0.379	0.627	0.222-1.773	0.334	1.568	0.63-3.902
Visceral	**0.015**	8.505	1.518-47.642	0.966	7.895	1.628-41.452	0.074	3.964	0.877-17.93	0.235	2.241	0.591-8.494
Bone	0.634	0.687	0.147-3.218	0.22	0.266	0.032-2.205	0.48	0.567	0.117-2.744	0.985	1.013	0.256-4.011
Age	0.721	0.993	0.954-1.033	0.365	0.968	0.901-1.039	0.653	0.99	0.947-1.035	0.532	0.985	0.939-1.033
BMI	**0.02**	0.784	0.639-0.963	0.226	1.239	0.876-1.750	0.623	0.952	0.784-1.157	**0.023**	1.257	1.032-1.53
PSA90	0.813	0.849	0.218-3.312	0.392	2.063	0.393-10.82	0.068	0.25	0.057-1.108	0.535	0.689	0.212-2.235
nPSA	0.314	1.736	0.593-5.081	0.622	1.479	0.312-7.021	**0.048**	3.322	1.008-10.94	0.211	2.428	0.605-9.745
TTN	0.111	0.915	0.82-1.021	**0.006**	1.274	1.072-1.515	**0.05**	0.89	0.791-1.00	0.218	1.063	0.964-1.173
PSA in the 3m	**0.003**	3.145	1.474-6.71	0.055	2.937	0.979-8.809	**0.007**	3.149	1.373-7.223	0.083	1.966	0.915-4.223
PSA in the 6m	0.73	0.839	0.31-2.269	**0.0001**	122.701	13.45- 1119.24	0.546	1.382	0.483.3.951	**0.0001**	13.995	4.238-46.214
PSA in the 12m	**0.0001**	24.74	5.727-106.874	**0.029**	11.573	1.281-104.59	**0.006**	7.147	1.746-29.25	0.566	1.587	0.328-7.673

Within-group comparisons were made using the bonferroni method with α` = 0.0083, with p < 0.0083 being considered significantly different, and the rest being considered significantly different at p < 0.05. nPSA: nadir PSA levels; TTN: time to nadir.

PSA90: PSA decreased to 90% from initial three months post-dose, PSA in the 3 month: PSA decreased to 0.2ng/ml at 3 months postdose, PSA in the 6 month: PSA decreased to 0.2ng/ml at 6 months post-dose, PSA in the 12 month: PSA decreased to 0.2ng/ml at 12 months post-dose,

Bold values represent statistically significant p-values.

**Figure 1:**
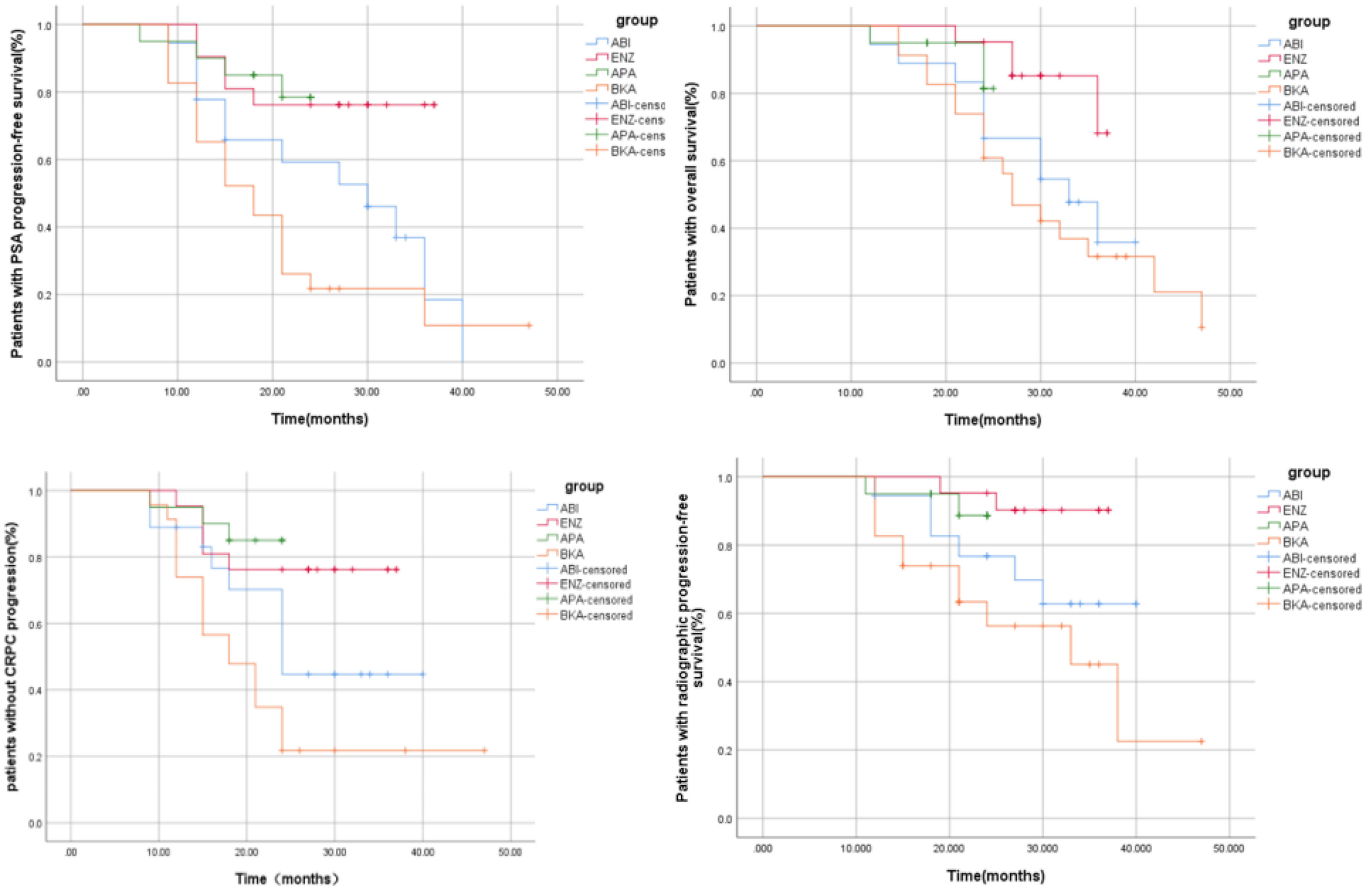
Survival curve.

Compared with bicalutamide, enzalutamide significantly prolonged patients’ rPFS (HR 2.539, 95% CI 1.1815.461, P= 0.007), and there was no significant difference in rPFS between the remaining three treatment groups. Upon analysis of the survival outcomes of the patients, it was discovered that enzalutamide significantly prolonged patient OS compared with bicalutamide (HR 2.06; 95% CI 1.183-3.585; P=0.005) (**Figure 1**). Furthermore, there was no notable distinction between the two remaining medications. The results of the COX multifactorial analysis showed that different mHSPC medications were the main factors influencing patients’ delayed disease progression (PSA-PFS: HR:1.107, 95% CI 0.712-1.724; P=0.041; rPFS: HR 0.469; 95% CI: 0.218-1.009; P=0.043) and OS (HR:0.541, 95% CI 0.329-.089; P=0.016).

### PSA kinetics

**Figure 2:**
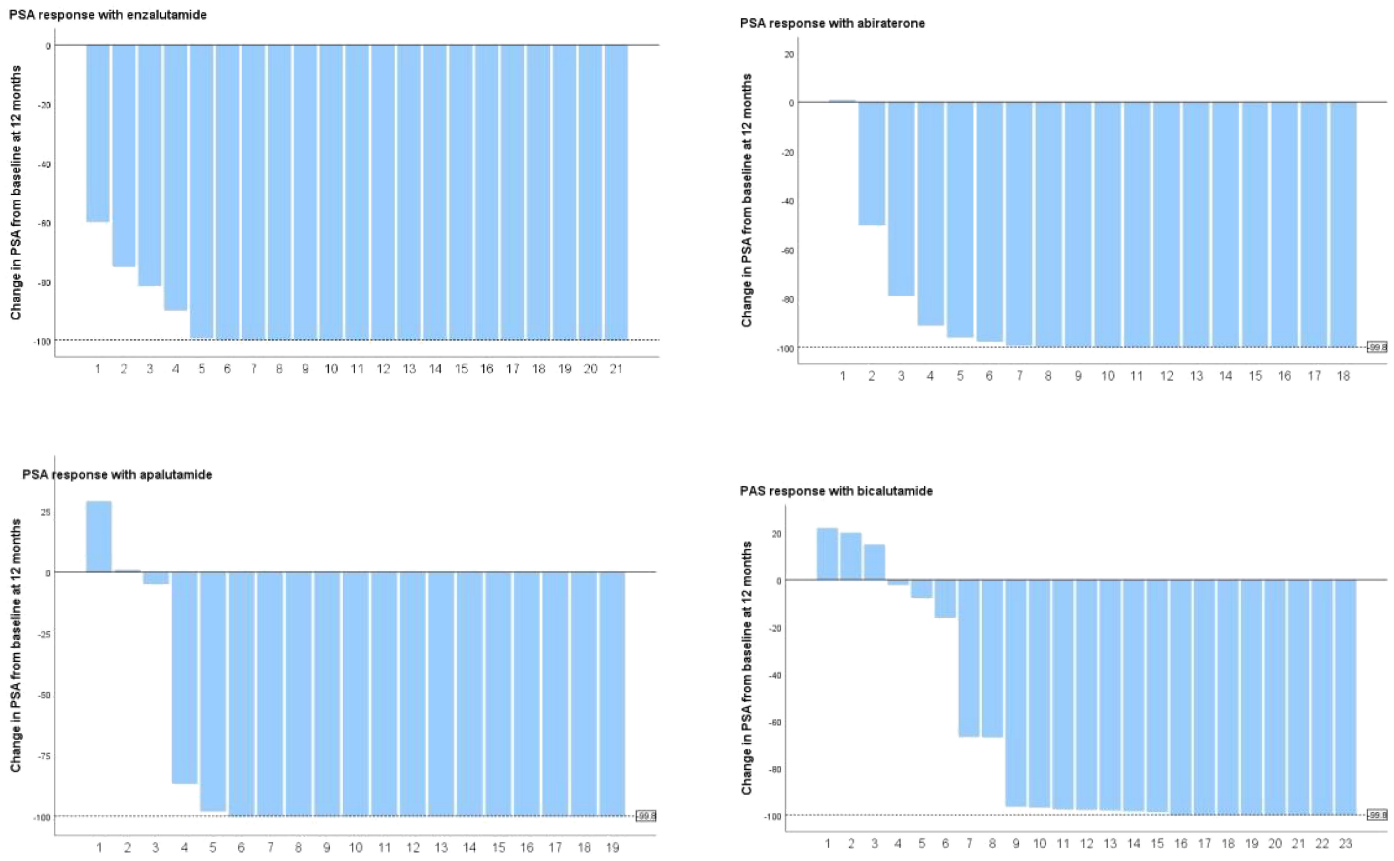
PSA response rate at 12 months after receiving the dose.

PSA levels did not show significant differences among the four groups at baseline (**Table 1**). After three months of treatment, 90.24% (74/82) of patients registered a 90% decrease in PSA, with enzalutamide proving to be significantly more effective than bicalutamide (100% vs. 73.9%, P=0.003) (**Table 4** and **Table 7** appendix). The remaining two treatment groups showed no significant difference. At 3 months after dosage, 43.9% of patients attained a deep PSA response (≤0.2ng/ml). Enzalutamide and apalutamide were found to be significantly more effective than bicalutamide (61.90% vs. 21.73%, p=0.007; 70.00% vs. 21.73%, p=0.005), whereas there was no statistically significant difference between abiraterone and the other three treatment groups, and this result was also observed at 6 months after dosage. After 12 months of receiving the dose (**Figure 2**), a larger percentage of patients attained deep PSA remission with abiraterone acetate, enzalutamide, and apalutamide as opposed to bicalutamide (34.8% vs 66.7% vs 76.2% vs 80.0%, p=0.007).

**Table 4:** PSA changes after drug administration.

	ABI (n=18)	ENZ (n=21)	APA (n=20)	Bica (n=23)	*P* value
PSA decline at landmark 3 months, PSA decline90%					**0.018**
yes	17 (94.4)	21 (100.0)	19 (95.0)	17 (73.9)	
no	1 (5.6)	0 (0.0)	1 (5.0)	6 (26.1)	
PSA decline at landmark 3 months, PSA ≤0.2ng/ml					**0.008**
yes	4 (22.2)	13 (61.9)	14 (70.0)	5 (21.7)	
no	14 (87.8)	8 (38.1)	6 (30.0)	18 (78.3)	
PSA decline at landmark 6 months, PSA ≤0.2ng/ml					**0.002**
yes	12 (66.7)	16 (76.2)	16 (80.0)	7 (30.4)	
no	6 (33.3)	5 (23.8)	4 (20.0)	16 (69.4)	
PSA decline at landmark 12 months, PSA ≤0.2ng/ml					**0.007**
yes	10 (66.7)	16 (76.2)	16 (80.0)	8 (34.8)	
no	8 (33.3)	5 (23.8)	4 (20.0)	15 (65.20)	
nPSA ≤0.2ng/ml					**0.014**
yes	11 (61.10)	18 (85.70)	17 (85.00)	11 (47.80)	
no	7 (38.90)	3 (14.30)	3 (15.00)	12 (52.20)	
nPSA (media,ng/ml)					**0.001**
	0.035	0.006	0.012	0.274	
TTN (media time,months)					0.471
	6	5	5.5	9	

Bold values represent statistically significant p-values.

In addition, enzalutamide and apalutamide had significantly shorter TTN than bicalutamide (6 months vs 5 months vs 5.5 months vs 9 months), although the difference was not statistically significant. Furthermore, there was a notable difference in nPSA concentrations within the four drug categories (0.035 vs. 0.006 vs. 0.012 vs. 0.274).

This indicates that enzalutamide and apalutamide have lower nPSA levels in contrast to bicalutamide (0.006 vs. 0.274, p=0.001; 0.012 vs. 0.274, p=0.006).

### Association between PSA deep response and outcomes

In our investigation, we observed significant benefits when the level of nPSA reached 0.2ng/ml or lower following medical therapy: significant improvement in PSA-PFS (HR: 10.783; 95%CI: 4.984-23. 329; a significant increase in overall survival (HR: 2.207; 95% CI: 1.115 - 4.365; p = 0.023), rPFS and CRPC-PFS (HR: 2.455; 95% CI: 1.036 - 5.82; p = 0.041, HR: 0.891; 95% CI: 0.813 - 0.977; p = 0.014) (**Table 3** appendix). Patients who attained PSA90 three months after the dose were significantly effective in extending PSA-PFS (HR: 2.395; 95% CI: 0.991-5.788; p=0.048) and r PFS (HR:5.775; 95% CI: 2.155-15.456; p=0.0001). At 3 months post-dose, patients who achieved a deep response in PSA also had a significant benefit in PSA-PFS (HR: 3.145, 95% CI: 1.474-6.71, p=0.003) and CRPC-PFS (HR: 3.149, 95% CI: 11.373-7.223, p=0.007). Patients who maintained a PSA level of <0.2ng/ml at 6 months post-dose demonstrated considerable benefits regarding prolonged OS (HR: 2.12; 95% CI: 2.47-12.149; p=0.0001), PSAPFS (HR: 1.83; 95% CI: 0.973-3.44; p=0.061), and r PFS (HR: 19.266; 95% CI: 4.476-82.929; p=0.0001). Patients who maintained PSA levels of ≤0.2ng/ml for 12 months after treatment demonstrated a substantial improvement in OS (HR: 19.886; 95%CI: 1.074-4.188; p=0.03), PSA-PFS (HR: 19. 701; p=0.0001), r-PFS (HR: 3.194; 95%CI:1.318-7.743; p=0.01) and CRPC-PFS (HR: 13.337; 95%CI: 5.714-31.134; p=0.0001).

### Adverse events

Upon recording adverse events during treatment (**Table 5**), it was found that there was no significant difference in the occurrence of overall adverse events among the four medicines (Grade I to II: 89.1% vs. 85.8% vs. 75.0% 91.3%; Grade III to IV: 38.9% vs. 38.1% vs. 35.5% vs. 39.1%). Bicalutamide heightened the risk of gynecomastia significantly more than the other three medication groups (17.4% vs. 5.0% vs. 4.8% vs. 11.1%) and induced a greater likelihood of rash (30.4%), hot flushes (47.8%) and weight loss (21.7%) than the other three groups. All four drug groups caused fatigue in patients to some extent. Abiraterone and enzalutamide had the highest incidence of fatigue (44.4% vs 42.9% vs 30.1% vs 26.1%). Abiraterone caused a greater number of adverse reactions than the other three treatment groups, mainly gastrointestinal, including nausea (22.2%), vomiting (33.3%), anorexia (44.4%), and pain, mainly arthralgia (22.2%) and bone pain (33.3%). Furthermore, it should be mentioned that enzalutamide was more likely to cause hypertension (28.6%) than the other three groups. The incidence of adverse events was slightly lower in the apalutamide group than in the other treatment groups.

**Table 5:** Adverse events.

	ABI (n=18)		EZA (n=21)		APA (n=20)		Bica (n=23)	
**Grade** (**%**)	I~II	III~IV	I~II	III~IV	I~II	III~IV	I~II	III~IV
**Any**	88.9 (16)	38.9 (7)	85.8 (18)	38.1 (8)	75.0 (15)	35.5 (7)	91.3 (21)	39.1 (9)
Hypertension	16.7 (3)	0	28.6 (6)	4.8 (1)	15.0 (3)	5.0 (1)	13.0 (3)	4.0 (1)
Hyperglycinemia	11.1 (2)	0	4.8 (1)	0	10.0 (2)	0	4.0 (1)	0
Skin rash	11.1 (2)	5.6 (1)	14.2 (3)	4.8 (1)	15.0 (3)	0	30.4 (7)	13.0 (3)
Peripheral oedema	33.3 (6)	0	19.0 (1)	0	10.0 (2)	0	4.0 (1)	0
Hot flush	11.1 (3)	0	23.8 (5)	14.2 (3)	5.0 (1)	0	47.8 (11)	17.4 (4)
Profuse sweating	27.8 (5)	0	9.5 (2)	4.8 (1)	10.0 (2)	0	8.7 (2)	0
Weight increased	5.6 (1)	0	9.5 (2)	0	10.0 (2)	5.0 (1)	4.0 (1)	0
Weight decline	5.6 (1)	5.6 (1)	9.5 (2)	4.8 (1)	10.0 (2)	0	21.7 (5)	4.0 (1)
Appetite decline	22.2 (4)	5.6 (1)	14.2 (3)	0	10.0 (2)	0	4.0 (1)	0
Constipation	16.7 (3)	0	9.5 (2)	0	5.0 (1)	0	17.4 (4)	4.0 (1)
Bloody stool	16.7 (3)	5.6 (1)	4.8 (1)	4.8 (1)	0	0	4.0 (1)	0
Vomiting	11.1 (2)	0	9.5 (2)	0	5.0 (1)	0	8.7 (2)	0
Nauseous	22.2 (4)	0	4.8 (1)	4.8 (1)	15.0 (3)	0	13.0 (3)	0
Diarrhea	33.3 (6)	5.6 (1)	23.8 (5)	4.8 (1)	10.0 (2)	0	8.7 (2)	4.0 (1)
Fatigue	44.4 (8)	5.6 (1)	42.9 (9)	4.8 (1)	30.1 (9)	0	26.1 (6)	13.0 (3)
Blood alkaline phosphatase increased	5.6 (1)	5.6 (1)	9.5 (2)	4.8 (1)	10.0 (1)	5.0 (1)	4.0 (1)	0
Anemia	5.6 (1)	0	14.2 (3)	0	5.0 (1)	5.0 (1)	8.7 (1)	0
Hypokalemia	5.6 (1)	0	9.5 (2)	4.8 (1)	10.0 (2)	5.0 (1)	0	0
Back pain	16.7 (3)	0	23.8 (5)	2	10.0 (2)	0	17.4 (1)	8.7 (2)
Arthralgia	22.2 (4)	5.6 (1)	9.5 (2)	4.8 (1)	15.0 (3)	5 (1)	8.7 (1)	0
Bone pain	33.3 (6)	11.0 (2)	19.0 (4)	2	10.0 (2)	5.0 (1)	13.0 (3)	8.7 (2)
Urine storage	5.6 (1)	0	4.8 (1)	0	5.0 (1)	5.0 (1)	8.7 (1)	0
Urinary tract infection	16.7 (3)	0	4.8 (1)	0	10.0 (2)	0	4.0 (1)	4.0 (1)
Frequent urination	11.1 (2)	0	14.2 (3)	0	15.0 (3)	5.0 (1)	8.7 (2)	0
Hematuria	11.1 (2)	0	4.8 (1)	0	5.0 (1)	5.0 (1)	8.7 (2)	4.0 (1)
Falling	5.6 (1)	1	9.5 (2)	4.8 (1)	10.0 (2)	0	4.0 (1)	0
Hypersomnia	11.1 (2)	0	4.8 (1)	0	5.0 (1)	0	17.4 (4)	8.7 (2)
Tachycardia	5.6 (1)	0	9.5 (2)	0	0	0	4.0 (1)	0
Dyspnea	11.1 (2)	5.6 (1)	4.8 (1)	0	5.0 (1)	0	8.7 (2)	0
Respiratory tract infection **Events of concern**	5.6 (1)	0	14.2 (3)	0	10.0 (2)	0	13.0 (3)	0
Gynecomastia	11.1 (2)	11.1 (2)	4.8 (1)	4.8 (1)	5.0 (1)	5.0 (1)	17.4 (4)	17.4 (4)
Fracture	11.1 (2)	11.1 (2)	9.5 (2)	9.5 (2)	10.0 (2)	10.0 (2)	4.0 (1)	4.0 (1)
Hypothyroidism	11.1 (2)	11.1 (2)	4.8 (1)	4.8 (1)	5.0 (1)	5.0 (1)	0	0

## Discussion

To our knowledge, this is the first study to compare the efficacy of multiple ARSIs used to treat patients with mHSPC. The purpose of this study was to address the current knowledge gap by analyzing the effectiveness variations of ARSIs, including abiraterone acetate, enzalutamide, apalutamide, and bicalutamide, in combination with ADT treatment for patients with mHSPC in an actual clinical setting to provide direct evidence-based support for the clinical use of these drugs.

Enzalutamide and apalutamide significantly slowed disease progression and prolonged survival in patients compared with bicalutamide in this real-world study. Significant association between OS and disease progression has been observed in prostate cancer patients with metastatic hormone-sensitive prostate cancer (mHSPC). The meta-analysis results demonstrated that effective prolongation of metastasis-free survival and time free of disease progression increased OS in patients with limited prostate cancer and those with mHSPC and non-metastatic desmoplastic resistant prostate cancer (nmCRPC) (16–18). Furthermore, Martini et al. conducted a supplementary analysis utilizing outcome data from the CHAARTED trial, which demonstrated that progression within 6 months of dosing had a noteworthy impact on patients’ overall survival (19).Therefore although our trial has not yet reached the median OS for apalutamide, analysis of the early progression-free survival data indicates that apalutamide significantly prolongs OS in mHSPC patients compared with bicalutamide.

In this study, abiraterone demonstrated no superiority in impeding the progression of the disease in comparison to alternative medication. Ueda and colleagues analyzed the factors affecting the prognosis of mHSPC patients treated with abiraterone and found that there was no significant difference between the effects of abiraterone and bicalutamide on the OS of patients with mHSPC overall, but that abiraterone significantly prolonged the OS of patients compared with bicalutamide in those with a Gleason major tissue score of < 5 (p = 0.0192) (20). In addition to the patient’s pathological characteristics having an impact on the drug’s effectiveness, variances in pharmacological action could also play a part in the difference in effectiveness between abiraterone and the other medications. Abiraterone inhibits the development and progression of PCa by irreversibly inhibiting CYP17A1 of the cytochrome P450 family, blocking androgen production (4). As inhibitors of the androgen receptor, enzalutamide and apalutamide possess the ability to bind specifically to the androgen receptor. This action prevents translocation, DNA binding androgen receptor-mediated transcription, resulting in the effective inhibition of prostate cancer cell proliferation. Enzalutamide and apalutamide demonstrate stronger receptor binding than bicalutamide and effectively suppress certain effects of activation (21).

In addition, abiraterone is often given with glucocorticoids, which significantly increases the likelihood of adverse gastrointestinal reactions in patients. As a result, many patients taking abiraterone to alleviate gastrointestinal symptoms also take proton pump inhibitors (PPIs) as an adjunct therapy. Studies have shown that PPIs have tumorigenic activity in prostate cancer cells and can stimulate the proliferation of hormone-sensitive cells. This may reduce the anti-tumor effect of abiraterone. Abiraterone is often used in combination with glucocorticoids, which significantly increases the likelihood of upper gastrointestinal side effects. As a result, many patients treated with abiraterone also use proton pump inhibitors (PPIs) as adjunctive therapy to relieve GI symptoms. However, studies have shown that PPIs have tumour-promoting activity in prostate cancer cells and can promote the proliferation of hormone-sensitive prostate cancer cells (22). A re-analysis of several large clinical trials found that the use of PPIs significantly reduced the therapeutic efficacy of abiraterone in patients with mHSPC (23). In an additional context, the use of glucocorticoids has the potential to alter the composition of the patient’s gut flora and microenvironment, which may also reduce the anti-tumour efficacy of abiraterone (24).

In light of the aforementioned reasons, we postulate that various factors may influence the therapeutic efficacy of abiraterone, resulting in its slightly inferior ability to delay disease progression compared to apalutamide and enzalutamide. This conclusion is corroborated by the results of a meta-analysis. The findings of the article suggest that the three novel endocrine agents (abiraterone, enzalutamide, and apalutamide) offer greater advantages in the treatment of mHSPC compared to other therapeutic approaches. Furthermore, apalutamide exhibits the most optimal efficacy, while enzalutamide is most effective in mHSPC patients with a low tumor burden. The therapeutic benefits of abiraterone are slightly inferior to those of the aforementioned drugs (25).

Changes in PSA levels are typically strongly linked to the prognosis of patients (26). Following a comparative analysis of PSA changes induced by four medicines, we found that enzalutamide, apalutamide, and abiraterone acetate had a significant advantage over bicalutamide in inducing deep PSA response (PSA ≤ 0.2 ng/ml) and that the effects of enzalutamide and apalutamide were faster. At 3 months after the first dose, we observed that a significantly higher proportion of patients taking enzalutamide and apalutamide achieved deep PSA remission (PSA ≤ 0.2 ng/ml) than those in the bicalutamide group (p=0.007, p=0.008). This result was observed equally at 6 and 12months postdose. Lowentritt and co-authors found that patients in the apalutamide group had a shorter median time to achieve deep PSA90 remission compared with abiraterone (3.5 months vs NR), and a greater number of patients achieved deep PSA response (27). In addition, we found that patients in the enzalutamide and apalutamide groups had a significantly higher proportion of nPSA in deep response than those in the bicalutamide group (85.7% vs. 47.80%, p=0.007; 85.0% vs. 47.80%, p=0.008) and a shorter TTN (5 months vs. 5.5 months vs. 9 months), while abiraterone acetate did not show a statistically significant difference. Results from a recent study showed that enzalutamide significantly increased PSA response rates in black patients compared with bicalutamide. In black patients, the PSA response rate after 7 months of treatment was significantly better in patients taking enzalutamide than in those taking bicalutamide (93% vs. 42%, P = 0.009). Enzalutamide had a significantly better PSA response rate than bicalutamide at 12 months (84% vs 34%) in the overall population (28).

Trial results have indicated that achieving deep nPSA remission (nPSA ≤ 0.2 ng/ml) and a longer TTN can be advantageous in terms of prolonging patient survival and delaying disease progression. The study revealed that both nPSA and TTN were significant prognostic factors for the progression to CRPC in patients with mHSPC. Furthermore, patients with nPSA levels of ≤ 0.2 ng/ml and TTN ≥ 9 months significantly delayed disease progression and prolonged OS (29, 30). However, there is no conclusive evidence to determine whether these two measures significantly improve or worsen patient outcomes, and their specific impact on patient prognosis remains unknown. In our trial, patients with nPSA≤ 0.2 ng/ml had better survival and disease progression (including PSA-PFS, rPFS, and CRPC-PFS) than those who did not, despite differences in the medicines used. Contrary to previous trials, the prognostic impact of TTN differed, whereby patients administered enzalutamide and apalutamide had a significantly shorter TTN compared with patients administered bicalutamide (5 months vs. 5.5 months vs. 9 months). It is hypothesized that this phenomenon may be attributable to previous trials neglecting to consider variances in patient medication preferences when seeking to lower PSA levels. Results from the TITAN trial show that patients with mHSPC treated with ADT alone achieved median nPSA levels of greater than 0.2 ng/mL, while patients in the apalutamide plus ADT group achieved nPSA levels of less than 0.02 ng/mL. In addition, the additional apalutamide to ADT prolonged patients’ TTN from 5 months to 6 months (5). Taking the above evidence together, we conclude that lower nPSA levels are more important for the prognostic impact on patients, and that achieving deep response with nPSA (PSA ≤ 0.02 ng/mL) is beneficial for delaying disease progression.

In recording adverse events caused by the four drugs during treatment, it was found that the incidence of adverse events caused by the four drugs was not significantly different overall and only for single adverse events. The results of a meta-analysis showed that among second-generation ARSIs, enzalutamide was associated with a significantly higher risk of increased blood pressure (SUCRA 0%) and headache (SUCRA 3%) in patients with mHSPC (31). Additionally, Zhang X and colleagues discovered that second-generation ARSIs were more effective than bicalutamide in prolonging both patient’s survival and disease progression, without a notable increase in the occurrence of adverse events (32). This aligns with our own findings in reaching these conclusions.

There are some limitations in this study. Firstly, due to the short time that Darolutamide has been available in China, the data were not strong enough to be included in the study for analysis. Secondly, bicalutamide combined with androgen deprivation therapy (ADT) is not currently employed as the first-line strategy for the treatment of mHSPC, but given the limited healthcare resources in certain regions, this combination therapy is still widely used and its clinical data are of some importance. Therefore, we opted to consider them for the investigation and utilize them as a control group for the experiment. Finally, the limited number of patients precluded the analysis of different subgroups and the exploration of specific discrepancies in the effectiveness of various medicines in different groups of patients. Despite the limitations of this study, we maintain that the trial results could substantially contribute to filling the present gap in knowledge regarding the efficacy and safety of various ARSIs in managing mHSPC patients.

This will provide a more reliable theoretical basis for the selection and use of medicines in patients with mHSPC.

## Conclusion

Abiraterone, enzalutamide, and apalutamide were found to significantly reduce and stabilize PSA levels in mHSPC patients more quickly and thoroughly than bicalutamide. Furthermore, enzalutamide and apalutamide were found to significantly prolong survival and delay disease progression in mHSPC patients compared with bicalutamide. It should be noted that abiraterone did not demonstrate a significant advantage in delaying disease compared with enzalutamide and apalutamide. After conducting drug toxicity analyses, it was determined that there were no significant differences among the four drugs.

## Data availability statement

The raw data supporting the conclusions of this article will be made available by the authors, without undue reservation.

## Ethics statement

The studies involving humans were approved by Ethics Committee of the Second Hospital of Lanzhou University. The studies were conducted in accordance with the local legislation and institutional requirements. Written informed consent for participation was not required from the participants or the participants’ legal guardians/next of kin because informed consent is not required for retrospective data collection. Written informed consent was obtained from the individual(s) for the publication of any potentially identifiable images or data included in this article.

## Author contributions

YL: Writing – review & editing, Writing – original draft, Visualization, Investigation, Conceptualization. JJ: Writing – review & editing, Validation, Supervision, Software, Methodology, Data curation. GY: Writing – review & editing, Project administration, Methodology, Formal analysis. ZW: Writing – review & editing. YW: Data curation, Writing – original draft. JW: Formal analysis, Writing – review & editing. ZZ: Data curation, Writing – original draft. LY: Supervision, Writing – review & editing. QZ: Resources, Supervision, Writing – review & editing. LJ: Data curation, Formal analysis, Writing – original draft. HD: Methodology, Writing – review & editing. YZ: Data curation, Writing – review & editing. ZD: Supervision, Writing – review & editing. JT: Project administration, Writing – review & editing.
