# Supplementary material for: Comparative effectiveness of multiple androgen receptor signaling inhibitor medicines with androgen deprivation therapy for metastatic hormone-sensitive prostate cancer: a study in the real world
Source: Front Oncol. 2024 Apr 18;14:1324181. doi: 10.3389/fonc.2024.1324181 (PMC11063352; doi:10.3389/fonc.2024.1324181)
Supplement: Supplementary file 1 [file Table_1.docx]

**Appendix: Comparison within group**

Table 6 COX analysis

| **Single factor analysis** | | |  |  |  |  |  |  |  |  |  |  |
| --- | --- | --- | --- | --- | --- | --- | --- | --- | --- | --- | --- | --- |
|  | **PSA-PFS** | | | **rPFS** | | | **CRPC-PFS** | | | **OS** | | |
|  | p value HR 95%CI | | | p value HR 95%CI | | | p value HR 95%CI | | | p value HR 95%CI | | |
| Totally | **0.029** | 1.289 | 0.242-1.528 | **0.042** | 1.399 | 0.947-2.067 | **0.044** | 1.371 | 0.995-1.89 | **0.026** | 1.203 | 0.892-1.623 |
| ABI VS ENZ | 0.038 | 0.324 | 0.112-0.939 | 0.083 | 0.242 | 0.049-1.205 | 0.112 | 0.411 | 0.138-1.228 | 0.06 | 0.326 | 0.102-1.048 |
| ABI VS APA | 0.231 | 0.686 | 0.371-1.24 | 0.356 | 0.67 | 0.287-1.566 | 0.058 | 0.531 | 0.276-1.023 | 0.333 | 0.71 | 0.355-1.421 |
| ABI VS BKA | 0.193 | 1.177 | 0.921-1.504 | 0.232 | 1.225 | 0.878-1.710 | 0.102 | 1.251 | 0.957-1.636 | 0.535 | 1.088 | 0.833-1.422 |
| ENZ VS APA | 0.831 | 0.866 | 0.233-3.228 | 0.453 | 2.51 | 0.227-27.791 | 0.51 | 0.618 | 0.148-2.587 | 0.234 | 3.965 | 0.411-38.248 |
| ENZ VS BKA | **0.001** | 2.244 | 1.366-3.685 | **0.007** | 2.539 | 1.181-5.461 | **0.003** | 2.131 | 1.295-3.506 | **0.005** | 2.06 | 1.183-3.585 |
| APA VS BKA | **0.003** | 5.071 | 1.711-15.032 | **0.06** | 4.367 | 0.941-20.265 | **0.002** | 6.724 | 1.976-22.87 | 0.168 | 2.506 | 0.678-9.258 |
| nPSA | **0.0001** | 10.783 | 4.984-23.329 | **0.041** | 2.455 | 1.036-5.82 | **0.014** | 0.891 | 0.813-0.977 | 0.023 | 2.207 | 1.115-4.365 |
| TTN | **0.033** | 0.912 | 0.838-0.993 | 0.134 | 1.076 | 0.978-1.185 | **0.0001** | 9.181 | 4.449-18.95 | 0.42 | 1.033 | 0.955-1.117 |
| PSA90 | **0.048** | 2.395 | 0.991-5.788 | **0.0001** | 5.772 | 2.155-15.456 | 0.321 | 1.616 | 0.627-4.166 | 0.168 | 1.88 | 0.767-4.609 |
| PSA in the 3m | **0.003** | 3.145 | 1.474-6.71 | 0.055 | 2.937 | 0.979-8.809 | **0.007** | 3.149 | 1.373-7.223 | 0.083 | 1.966 | 0.915-4.233 |
| PSA in the 6m | 0.061 | 1.83 | 0.973-3.44 | **0.0001** | 19.266 | 4.476-82.929 | 0.095 | 1.76 | 0.906-3.419 | **0.0001** | 5.478 | 2.47-12.149 |
| PSA in the12m | **0.0001** | 19.886 | 7.503-52.701 | **0.01** | 3.194 | 1.318-7.743 | **0.0001** | 13.337 | 5.714-31.13 | **0.03** | 2.12 | 1.074-4.188 |

Table 7 Comparative results within groups.

|  | **ABI** | **ENZ** | **APA** |
| --- | --- | --- | --- |
| **PSA90 in the**  **3th month** | ***p value*** | ***p value*** | ***p value*** |
| ABI | / |  |  |
| ENZ | 0.462 | / |  |
| APA | 1.000 | 0.448 | / |
| BKA | 0.067 | **0.003** | 0.049 |
| **PSA deep response in the 3th month** | | |  |
| ABI | / |  |  |
| ENZ | 0.023 | / |  |
| APA | 0.182 | 0.278 | / |
| BKA | 1.000 | **0.007** | **0.005** |
| **PSA deep response in the 6th month** | | |  |
| ABI | / |  |  |
| ENZ | 0.042 | / |  |
| APA | 0.023 | 0.768 | / |
| BKA | 0.355 | **0.002** | **0.001** |
| **PSA deep response in the 12th month** | | |  |
| ABI | / |  |  |
| ENZ | 0.723 | / |  |
| APA | 0.351 | 0.768 | / |
| BKA | **0.003** | **0.006** | **0.003** |
| **nPSA≤0.2ng/ml** | | |  |
| ABI | / |  |  |
| ENZ | 0.077 | / |  |
| APA | 0.144 | 0.948 | / |
| BKA | 0.397 | **0.007** | **0.008** |
| **nPSA** |  |  |  |
| ABI | / |  |  |
| ENZ | 0.054 | **/** |  |
| APA | 0.210 | 0.481 | / |
| BKA | 0.185 | **0.001** | **0.006** |
